# Hydroxyapatite Decorated with Tungsten Oxide Nanoparticles: New Composite Materials against Bacterial Growth

**DOI:** 10.3390/jfb13030088

**Published:** 2022-06-24

**Authors:** Francesca Silingardi, Francesca Bonvicini, Maria Cristina Cassani, Raffaello Mazzaro, Katia Rubini, Giovanna Angela Gentilomi, Adriana Bigi, Elisa Boanini

**Affiliations:** 1 Department of Chemistry ‘‘Giacomo Ciamician”, University of Bologna, Via Selmi 2, 40126 Bologna, Italy; francesca.silingard3@unibo.it (F.S.); katia.rubini@unibo.it (K.R.); adriana.bigi@unibo.it (A.B.); 2 Department of Pharmacy and Biotechnology, University of Bologna, Via Massarenti 9, 40138 Bologna, Italy; francesca.bonvicini4@unibo.it (F.B.); giovanna.gentilomi@unibo.it (G.A.G.); 3 Department of Industrial Chemistry “Toso Montanari”, University of Bologna, Viale del Risorgimento 4, 40136 Bologna, Italy; 4 Department of Phisics and Astronomy “A. Righi”, University of Bologna, Viale Berti Pichat 6/2, 40138 Bologna, Italy; raffaello.mazzaro@unibo.it

**Keywords:** calcium phosphate, bone, metal oxide nanoparticles, transmission electron microscopy, antibacterial activity, biomaterial

## Abstract

The availability of biomaterials able to counteract bacterial colonization is one of the main requirements of functional implants and medical devices. Herein, we functionalized hydroxyapatite (HA) with tungsten oxide (WO_3_) nanoparticles in the aim to obtain composite materials with improved biological performance. To this purpose, we used HA, as well as HA functionalized with polyacrilic acid (HAPAA) or poly(ethylenimine) (HAPEI), as supports and polyvinylpyrrolidone (PVP) as stabilizing agent for WO_3_ nanoparticles. The number of nanoparticles loaded on the substrates was determined through Molecular Plasma-Atomic Emission Spectroscopy and is quite small, so it cannot be detected through X-ray diffraction analysis. It increases from HAPAA, to HA, to HAPEI, in agreement with the different values of zeta potential of the different substrates. HRTEM and STEM images show the dimensions of the nanoparticles are very small, less than 1 nm. In physiological solution HA support displays a greater tungsten cumulative release than HAPEI, despite its smaller loaded amount. Indeed, WO_3_ nanoparticles-functionalized HA exhibits a remarkable antibacterial activity against the Gram-positive *Staphylococcus aureus* in absence of cytotoxicity, which could be usefully exploited in the biomedical field.

## 1. Introduction

The last tens of years have recorded a continuously increasing life expectancy in developed countries. Although this is generally considered a welcome phenomenon, it has the undesirable consequence of age-related health problems. In the biomedical field, it means a huge need for biomaterials, especially for orthopaedic implants. The substitution/repair of damaged tissues is too often accompanied by early and/or delayed, as well as late, infections [1,2]. Many factors contribute to the relatively high frequency of nosocomial infections [3,4]. In particular, a key role is played by the spreading of multidrug-resistant bacteria caused by the excessive use and abuse of antibiotics [5]. 

Among the number of strategies developed to overcome these problems, an outstanding approach is functionalization of biomaterials with antibacterial agents. In particular, inorganic nanoparticles are of great interest because of their reduced dimensions and strong reactivity, and they are utilized in a variety of biomedical applications, including as antibacterial agents [2,6,7]. Inorganic nanoparticles with antibacterial properties involve metals, such as Ag and Cu nanomaterials, which display several adverse side effects, such as toxicity to mammalian cells [8,9]. Metal oxide nanoparticles, such as TiO_2_ and ZnO, are less toxic and display photocatalytic properties, so under the stimulation of light, they produce reactive oxygen species, which can cause germ death [10]. The applications of WO_3_, a n-type semiconductor, spread from micro- and opto-electronics, to photocatalysis to bacterial degradation [11,12,13]. The stable phase of WO_3_ crystals at room temperature is monoclinic (γ-WO_3_), whereas further crystalline phases can be obtained through heat treatment [14,15]. WO_3_ nanoparticles do not exhibit a very strong antimicrobial activity, but their efficiency is significantly improved when combined with different materials [11]. It has been reported that WO_3_ nanoparticles stabilized by polyvinylpyrrolidone (PVP) display a significant cytotoxic effect on osteosarcoma cells in vitro, and no, or just very small, toxicity on normal cells [16]. Moreover, incorporation into polymer fibers was shown to provide materials able to reduce microbial growth of Gram-positive bacteria and the DNA virus [17]. 

Herein, we investigated the possibility to use hydroxyapatite (HA) nanocrystals as support for WO_3_ nanoparticles. HA is considered the most suitable calcium phosphate for the preparation of biomaterials for hard tissues repair, due to its high stability, excellent biocompatibility and bioactivity. The applications of HA, either alone or in combination with different materials, spread from coatings, to cements, to scaffolds for regenerative medicine [18]. We have previously demonstrated that HA nanocrystals can act as support for silver, as well as for platinum nanoparticles [19,20,21]. Composites based on HA and WO_3_ nanoparticles might improve the excellent biological performance of hydroxyapatite through addition of antibacterial properties. To this aim, we submitted HA nanocrystals, as well as HA functionalized with polyacrilic acid (HAPAA) or poly(ethylenimine), (HAPEI), to interaction with PVP-stabilized WO_3_ nanoparticles.

## 2. Materials and Methods

### 2.1. Materials Synthesis

All reagents and solvents were used as received; ultrapure water purified with the Milli-Q plus system (Millipore Co, resistivity over 18 MΩ cm) was used.

Hydroxyapatite (HA) was obtained through a precipitation synthesis, as previously reported [21], through dropwise addition of (NH_4_)_2_HPO_4_ (50 mL, 0.65 M) to Ca(NO_3_)_2_ (50 mL, 1.08 M) in N_2_ atmosphere, at pH = 10 and 90 °C. After the addition, the solution was kept at 90 °C under magnetic stirring for 5 h during which the pH was maintained at about 10 with NH_3_. The solution was then centrifugated at 10.000 rpm for 10 min. The precipitate was washed 3 times with CO_2_-free water and dried at 37 °C.

Poly(ethylenimine) and polyacrylic acid containing hydroxyapatite (HAPEI and HAPAA) were obtained following the synthesis procedure of HA, but with slight modifications: in the first case PEI (Sigma-Aldrich, St. Louis, MO, USA, MW~2000 g mol^−1^) was added to the cationic solution to give 8 M concentration in monomer; whereas in the second case, PAA (Sigma-Aldrich, MW~1800 g mol^−1^) at a concentration of 0.1 M in monomer was added to the anionic solution.

The synthesis of tungsten oxide nanoparticles was carried out by a slight modification of a literature method [16]. Briefly, 200 mL of a sodium tungstate solution (Na_2_WO_4_∙2H_2_O, 1.649 g, 0.005 mol, Sigma-Aldrich, ≥98 %) was passed through a column containing a strongly acidic cation exchange resin Amberlite^®^ IR120 (51.0 g) and added dropwise to a flask containing polyvinylpyrrolidone (PVP-10, Merck, Darmstadt, Germany, 9003–39-8, (C_6_H_9_NO)_x_, 111.1 g/mol, av. MW 10,000). The solution was stirred for 4 h under reflux to give a clear yellow solution.

For adsorption of WO_3_ nanoparticles, 500 mg of substrate (HA, HAPEI, HAPAA) were incubated with various volumes of WO_3_ colloidal solution (5, 20, 40, 80 and 120 mL). The suspensions were maintained at room temperature under magnetic stirring for 1 h. At the end of the incubation time, the suspensions were filtered on a Buchner funnel, and the obtained solids were washed with the minimum amount of H_2_O and dried at 37 °C. The resulting samples were labeled HAX, HAPAAX and HAPEIX, where X indicates the volume (number of mL) of the colloidal solution. They all appear as white powders (Figure S1).

### 2.2. Characterization

X-ray powder diffraction was carried out by means of a PANalytical X’Pert PRO powder diffractometer equipped with a fast X’Celerator detector. The Kα copper radiation (λ = 1.5418 Å) selected by a Ni filter was used as incident radiation. The working current and voltage values were 40 mA and 40 kV, respectively. For phase identification, the 2θ range was investigated from 3 to 60 degrees with a step size of 0.1° and time/step of 100 s. 

Further X-ray power diffraction data were recorded in two different regions of 2θ, namely 25–28° and 38–41°, with a step size of 0.008° and time/step of 150 s and utilized to evaluate the coherence length of the perfect crystalline domains (*τ_hkl_*), calculated using the Scherrer equation:

(1) $$\tau_{hkl}=\frac{K\;\lambda }{\beta_{1/2}\;cos\theta }$$

where *λ* is the wavelength, *θ* the diffraction angle and *K* a constant depending on crystal habit (chosen as 0.9).

Morphological investigation was performed by a Zeiss Leo-1530 (Zeiss, Milan, Italy) scanning electron microscope (SEM) equipped with InLens detector and operating at 1 kV. No sample coating was performed.

Microstructural and nanoscale compositional analyses were carried out by using a Philips TECNAI F20 ST transmission electron microscope (TEM) operating at 120 kV. High resolution TEM (HR-TEM) images were taken in the phase contrast mode. STEM micrographs were recorded using a High Angle Annular Dark Field (HAADF) detector. For TEM observations, powder suspensions in 2-propanol were sonicated and drop-casted on a copper grid coated with an amorphous carbon film.

The polymer (PEI or PAA) content of the substrate was measured through thermogravimetric analysis using a Perkin Elmer TGA-7 (Perkin Elmer, Milan, Italy). Samples (5–10 mg) were heated in a platinum crucible in N_2_ flow (20 cm^3^ min^−1^) at a rate of 10 °C min^−1^ up to 800 °C.

The overall amount of tungsten present on the different samples was determined by means of Agilent 4210 (Agilent, Santa Clara, CA, USA) Molecular Plasma-Atomic Emission Spectroscopy (MP-AES). Tungsten lines at 400.871 and 429.461 nm were used. The analyses were conducted by comparison with five calibration standards (2, 4, 6, 8, 10 ppm), prepared by dilution to 50 mL of a 100-ppm tungsten standard (prepared dissolving 17.7 mg of Na_2_WO_4_∙2H_2_O in a 100 mL flask with water). The samples were analyzed after treating the solids (ca. 10 mg) with 1.0 M nitric acid (Normatom^®^, VWR International, Milan, Italy) in 50 mL flasks and sonicated until complete dissolution. Results from this analysis represent the mean value of three different determinations. 

Zeta potential was measured using Electrophoretic Light Scattering (ZetasizerNano; Malvern Instruments Ltd., Malvern, UK). A total of 5 mg of powder sample was suspended in 50 mL of MilliQ water and sonicated for 2 min before zeta potential measurement. Each analysis was performed in triplicate.

WO_3_ release was determined on weighted samples maintained in physiological solution (0.9% NaCl) at 37 °C. A total of 1 mL of solution was added to 50 mg of sample and the whole liquid was substituted at each time point (t = 4, 8, 24 and 48 h). Results from this analysis represent the mean value of two different determinations. 

### 2.3. Antibacterial Activity

*Staphylococcus aureus* (ATCC 25923) and *Escherichia coli* (ATCC 25922) were selected as representative Gram-positive and Gram-negative bacterial models, respectively, to test the antibacterial properties of the composite biomaterials. The reference strains were purchased from the American Type Culture Collection (ATCC, Manassas, VA, USA), andwere routinely cultured in 5% blood agar at 37 °C. For experiments, the bacterial inocula were prepared in PBS (phosphate buffer saline), adjusted at 0.5 McFarland, corresponding to 10^8^ CFU (colony forming units)/mL, and subsequently diluted in PBS to the concentration of 10^3^ CFU/mL. To evaluate the antibacterial activity, the powder samples were added to the melted Mueller-Hinton agar II (Biolife Italiana, Milan, Italy), at different concentrations, and under stirring conditions. Once the agar reached the 55 °C, the homogeneous solutions was poured into Petri dishes (Ø = 90 mm). After cooling, 100 μL of bacterial solution was plated on the solidified agar, containing the samples at different amounts of WO_3_ and without (unloaded samples) as control [2,22]. Plates were incubated at 37 °C for 24–48 h to allow bacterial growth and thereafter imaged using the VersaDoc Image System. Colonies were digitally counted using the colony counting software ( Quantitation Software Version 4.6.9, Quantity One, Bio-Rad Laboratories, Inc., Hercules, CA, USA).

### 2.4. In Vitro Cytotoxicity and Cell Viability Assays

Vero cell line (ATCC CCL-81) was used to investigate the overall effect of the material samples on mammalian cells, by means of two different bioassays. In particular, the safety profile was quantitatively evaluated by measuring both cell viability and lactate dehydrogenase enzyme (LDH) release from damaged plasma membranes after the incubation with all the produced biomaterials.

**Figure 1:**
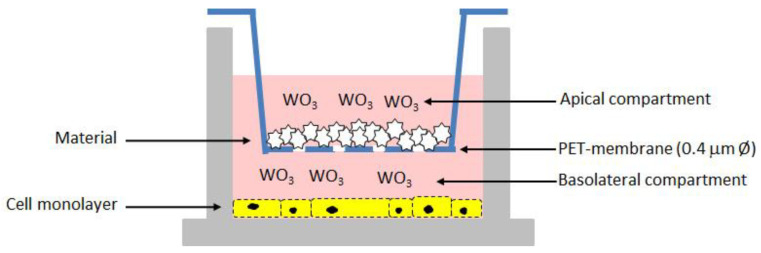
Schematic representation of the in vitro test for the cytotoxicity assessment on the composite biomaterials.

Briefly, cells were grown in RPMI-1640 medium supplied with 10% FBS (Fetal Bovine Serum), 100 U/mL penicillin, and 100 µg/mL streptomycin), at 37 °C and 5% CO_2_. For experiments, cells were seeded onto a 12-well tissue culture plate at a concentration of 7 × 10^4^ cells/well. Following 24 h of incubation, the 12-well cell culture inserts with a pore size 0.4 μm PET (polyethylene terephthalate)-membrane were used to expose cells to the different composite materials. Fresh regular medium was replaced in the basolateral compartment while medium containing the samples loaded with different amounts of WO_3_, and unloaded as control, was added in the apical compartment (Figure 1). After 48 h of incubation, the basolateral medium was recovered while the apical medium containing the biomaterials powder as well as the inserts were discarded. The cell viability and proliferation of Vero monolayer was assessed by using the CCK-8 solution (CCK-8, Cell Counting Kit-8, Dojindo Molecular Technologies, Munich, Germany) [23]. The OD values at 450 nm were measured after 2 h of incubation and data were expressed as percentage values of cell viability relative to the untreated control. In addition, the basolateral medium was assayed for the LDH release by using the Cytotoxicity LDH Assay Kit-WST (Dojindo Molecular Technologies). The measurement of the amount of released enzyme from cells is one of the major methods to assess the cell death. The OD values at 490 nm were measured and used to calculate the cytotoxicity (%) relative to lysed positive control.

### 2.5. Statistical Analysis

All biological assays were carried out in triplicate, and in three independent assays. One-tailed t test was used to compare the antibacterial activity of the pristine composite materials and their corresponding loaded samples. One-way analysis of variance (One-way ANOVA) followed by Dunnett’s Multiple Comparison Test was used to assess significant differences among samples loaded with different contents of WO3 and to compare samples at different concentrations. Differences were considered statistically significant with *p*-value < 0.05.

## 3. Results and Discussion

### 3.1. Materials Characterization

In this work, PVP-coated WO_3_ nanoparticles sols were synthesized through a recently reported ion exchange technique [16,24]. The UV-Vis spectrum, which shows an intense band at λmax = 325 nm, as well as the hydrodynamic diameter (Figure S2) and zeta potential (ca. −7 mV) data, were in keeping with what was reported in the literature [16].

**Figure 2:**
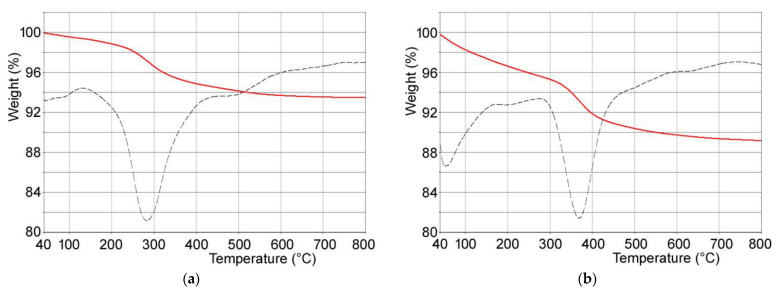
TG-DTG plots of (**a**) HAPEI and (**b**) HAPAA.

Composite materials were obtained through interaction of PVP-stabilized tungsten oxide nanoparticles with calcium phosphates crystals. In particular, (i) hydroxyapatite, (ii) poly(ethylenimine)-functionalized hydroxyapatite and (iii) polyacrylic acid-functionalized hydroxyapatite were used as substrates in order to modulate WO_3_ nanoparticles content of the composites. Polymer content of the functionalized hydroxyapatites supports was determined through thermogravimetric analysis: both PAA and PEI undergo complete combustion within 800 °C. In particular, the thermogravimetric plots of HAPAA and HAPEI (Figure 2) show that PAA and PEI decomposition occurs between 300 and 600 °C and between 150 and 700 °C, respectively, revealing polymer contents of about 6.3 ± 1 wt% and 5.8 ± 1 wt%.

**Figure 3:**
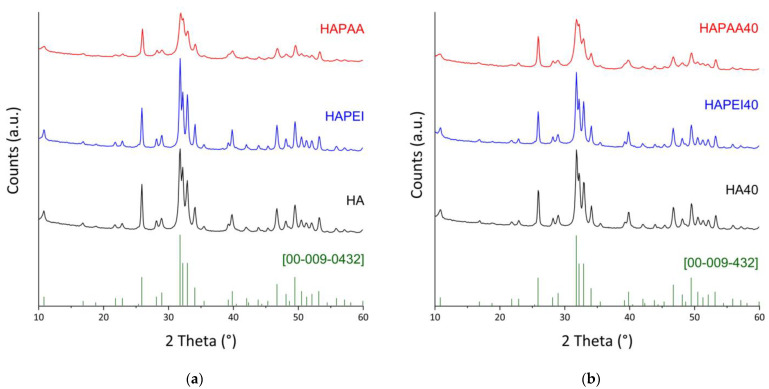
X-ray powder diffraction patterns of (**a**) the pristine supports; (**b**) the composite materials obtained after interaction with 40 mL of the WO_3_ colloidal suspension. The reference lines of HA (ICDD file 009–0432) are reported.

All of the samples, both before and after the interaction with WO_3_ nanoparticles, are constituted of hydroxyapatite as the only crystalline phase (Figure 3). Indeed, the peaks positions always match with ICDD PDF 009–0432. The presence of the polymers influences the crystallinity of the hydroxyapatite nanocrystals, in agreement with the different sharpness of the reflections in the XRD patterns reported in Figure 3. The effect of PAA is opposite to that of PEI: the mean dimensions of the perfect crystalline domains of HAPAA are smaller than those measured for HA, whereas PEI causes an increase in crystallinity in agreement with the bigger dimensions of τ_hkl_ values along both the direction parallel to the *c*-axis and that orthogonal to it (Table 1).

**Figure 4:**
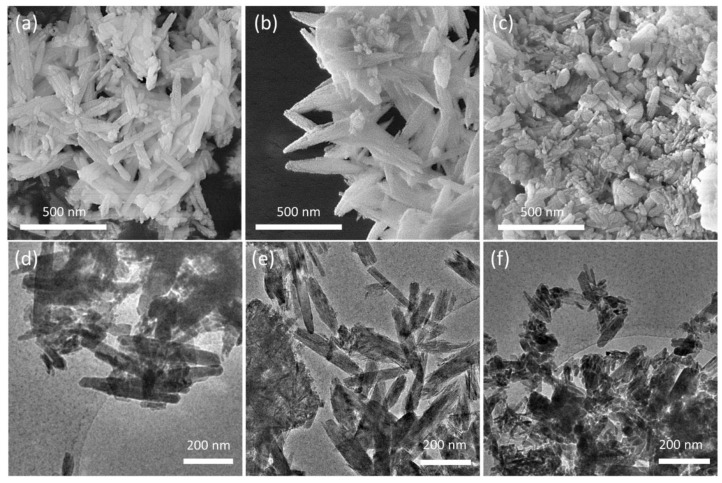
SEM images of (**a**) HA, (**b**) HAPAA and (**c**) HAPEI crystals and corresponding low-magnification TEM micrographs (**d**–**f**).

The presence of the polymers also influences the morphology of the nanocrystals [25,26], as shown by the comparison of the SEM and TEM images of the different substrates reported in Figure 4. HA crystals show the typical rod-like morphology, with mean dimensions ranging between 50–100 nm and 100–300 nm; HAPAA crystals appear jagged and exhibit pointed tips, as if they were exfoliated, whereas HAPEI crystals’ morphology is less defined, with thicker and more rounded rods alternated to smaller nanoparticles with less-defined size ratio.

The XRD patterns of the samples obtained after WO_3_ adsorption on the substrates do not show any evidence of the presence of the nanoparticles on the substrates (Figure 3). Loading on HA was explored up to a volume of WO_3_ nanoparticles colloidal suspension of 120 mL because this substrate gave composites with the best antibacterial properties (see Section 3.2). Moreover, the patterns of these composites do not show appreciable variations as a function of the presence of the nanoparticles (Figure S3), which do not significantly affect the dimensions of the perfect crystalline domains (Table 1).

**Figure 5:**
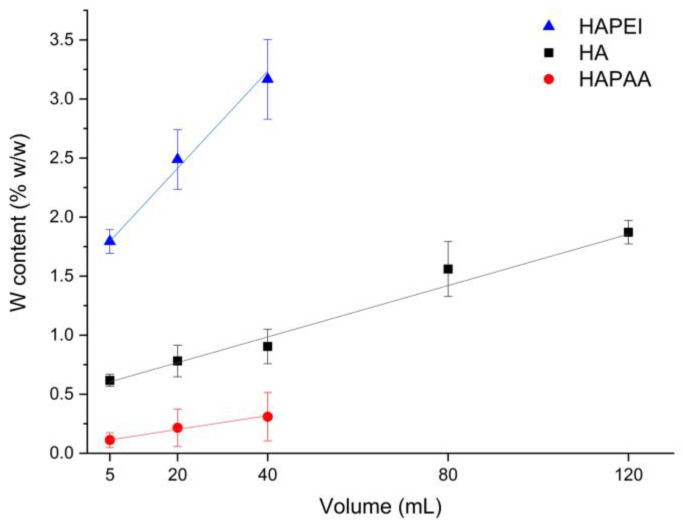
Tungsten amount loaded on the different supports through interaction with different volumes of the WO_3_ colloidal suspension.

The amount of tungsten oxide nanoparticles loaded on the substrates is indeed quite modest. The results of MP-AES analysis allow us to evaluate the number of adsorbed nanoparticles, which varies on the different substrates: the samples incubated with 40 mL of colloidal suspension exhibit an overall content of tungsten of about 0.3, 0.9 and 3.2 wt% on HAPAA, HA and HAPEI, respectively, whereas HA120 reaches a tungsten content of about 1.9 wt% (Figure 5). The greater number of nanoparticles loaded on HAPEI compared to the other substrates is not surprising since PVP-stabilized tungsten oxide nanoparticles have negative zeta-potential [15] and therefore are greatly attracted by the positively charged HAPEI. The attraction is reduced towards HA, and even more towards HAPAA, which display negative zeta potentials of −11.3 and −21.0 mV, respectively. The values of zeta potential of the composite materials decrease upon increasing the number of loaded nanoparticles, as reported in Table 1. 

**Figure 6:**
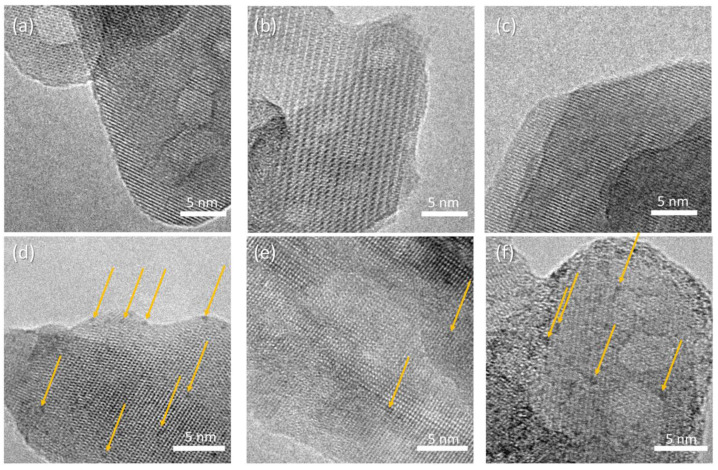
HR-TEM micrographs of (**a**) HA, (**b**) HAPAA, (**c**) HAPEI, (**d**) HA40, (**e**) HAPAA40, (**f**) HAPEI40. Highlighted by arrows, WO_3_ nanoparticles appearing as darker spots.

HR-TEM micrographs of the composite samples are reported in Figure 6, displaying crystalline rods supporting a homogeneous decoration with high-contrast sub-nm nanoparticles. The precise size distribution analysis is prevented by the lack of clear edges due to the superposition of the supporting HA crystals. The concentration of nanoparticles depends on the polymer addition, with the following trend: HAAPEI40 > HA40 > HAPAA40. The supporting rod structures display reflection patterns compatible with hydroxyapatite phase, for all samples (Figure S4). 

**Figure 7:**
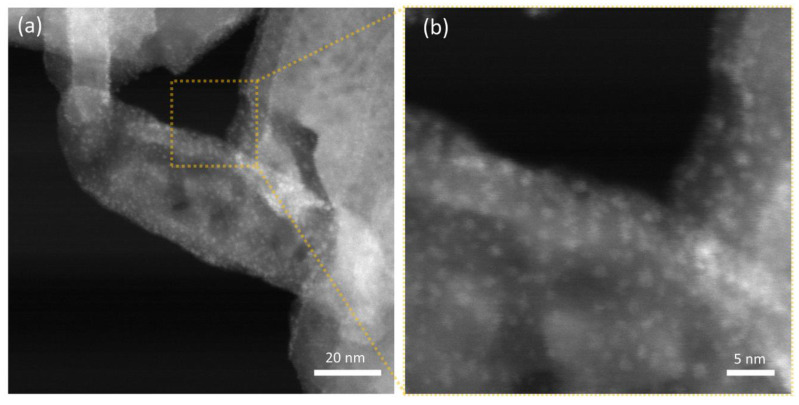
STEM-HAADF micrographs of (**a**) HAAPEI40 sample and (**b**) higher magnification of a single crystal functionalized with a uniform coating of WO_3_ nanoparticles, here appearing as brighter spots.

Thanks to the notable difference in scattering cross-section for the W-based nanoparticles and the hydroxyapatite nanostructures, STEM-HAADF micrographs allow for better visualization of the decoration, as demonstrated in Figure 7 for HAAPEI40 sample. This nanoscale characterization fully confirms the successful production of extremely disperse sub-nm nanoparticles, as well as loading of HA with such metal oxide nanostructures.

**Figure 8:**
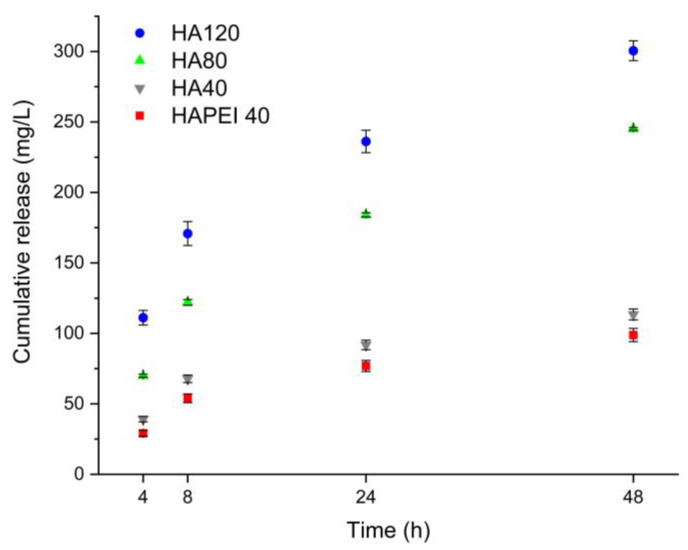
Tungsten cumulative release from the different supports in physiological solution.

Figure 8 reports the cumulative release of WO_3_ nanoparticles from HAPEI and HA in physiological solution. In spite of the higher nanoparticles content of HAPEI40, its cumulative release is even lower than that from HA40, confirming the strong interaction between PEI and the nanoparticles. Release increases with time so that after 48 h, it reaches values of about 110, 240 and 300 mg/L from HA40, HA80 and HA120, respectively.

### 3.2. Antibacterial Activity

To evaluate the antibacterial activity of the material samples, a standard colony-counting assay was employed; agar plates containing the proposed biomaterials at different concentrations were prepared, and 10^2^ CFU were spread on the agar surface. Bacterial cells were allowed to grow for 24–48 h at 37 °C, and then colonies were digitally counted and analyzed. Data were expressed as percentage values of CFU relative to bacterial cells grown on regular agar plates.

In a preliminary series of experiments, different materials (HA, HAPEI, HAPAA, HA40, PEI40, and PAA40) were assayed for their antimicrobial properties in order to identify the most suitable support for the active compound.

**Figure 9:**
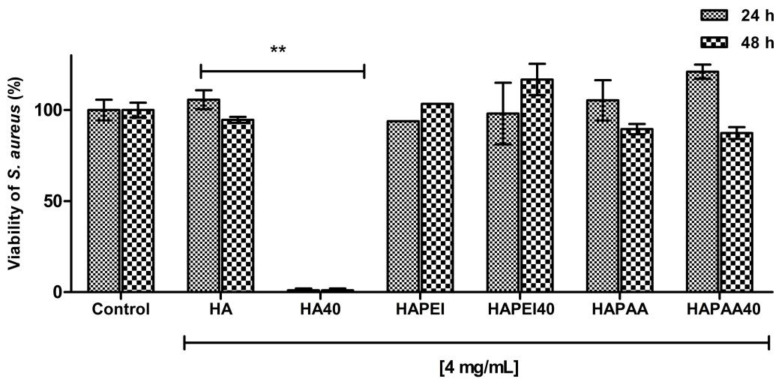
Antibacterial activity of the composite materials against *S. aureus* after 24 h and 48 h of incubation. Data are expressed as percentage values of the bacterial growth relative to the positive control. Error bars represent standard deviation. HA sample loaded with WO_3_ significantly decreases the viability of *S. aureus* (** *p* < 0.001).

As detailed in Figure 9, HA results to be the optimal material to support WO_3_ because the pristine material did not interfere with bacterial growth and the addition of the nanoparticles completely inhibited *S. aureus* growth as no detectable colonies were present following 24 and 48 h of incubation. This result is in agreement with the release data: although W content of HA40 is less than one third of that of HAPEI40, the relative amount of tungsten released from HA40 is indeed greater than that released from HAPEI40 (Figure 8). As a consequence of the strong interaction between PEI and WO_3_, the active compound does not diffuse into the medium, giving ineffective results against bacterial growth.

The effectiveness of the different composite materials was tested also against *E. coli*, using the same experimental approach. None of the tested biomaterials proved to be active on this Gram-negative strain. This result can be ascribed to the lower permeability of *E. coli* compared to the tested *S. aureus*, due to the presence of the outer membrane and lipopolysaccharides in the Gram-negative bacterial cells [27,28]. The highest antibacterial activity of WO_3_ nanoparticles against Gram-positive bacteria has been previously reported [17,29].

Further investigations were carried out on *S. aureus* aimed at determining the potency of HA samples loaded with different amounts of WO_3_; moreover, experiments were carried out by adding different concentrations of the composite materials to the agar plates in order to evaluate the minimum amount of WO_3_ to be loaded on HA as well as the minimum concentration of the whole biomaterials.

**Figure 10:**
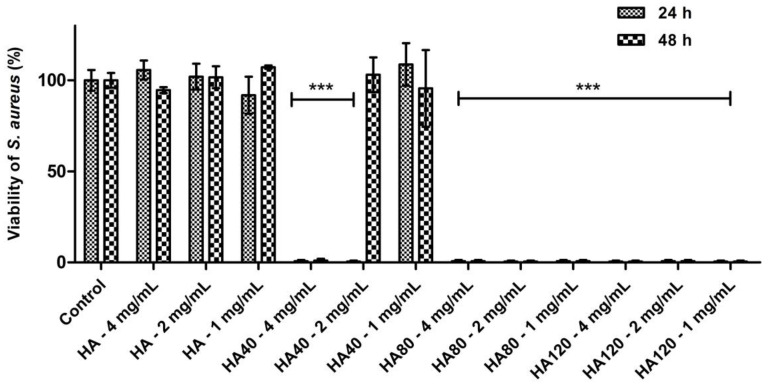
Antibacterial activity of HA and WO_3_ in different concentrations against *S. aureus* after 24 h and 48 h of incubation. Data are expressed as percentage values of the bacterial growth relative to the positive control. Error bars represent standard deviation. Differences were observed between HA and the corresponding samples when loaded with different amounts of WO_3_ (*** *p* < 0.0001).

As detailed in Figure 10, HA80 and HA120 effectively inhibited bacterial growth irrespective of the concentrations of biomaterials added to the agar medium; on HA40, the potency of the antibacterial activity was strictly related to the amount of the materials, thus indicating a dose-dependent effect of WO_3_ content on bacterial proliferation. As previously demonstrated, HA40 at 4 mg/mL prevented bacterial replication; indeed, no colonies were detected at 24 h and 48 h of growth. At lower concentrations, the bacterial proliferation was significantly affected: at 2 mg/mL, CFU were identified on the surface of the agar plates only after 48 h of incubation, suggesting a remarkable slowdown in the bacterial metabolism.

**Figure 11:**
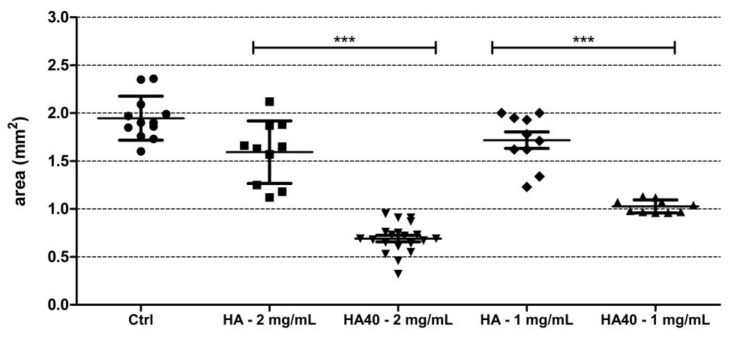
Graph showing the size (in mm^2^) of *S. aureus* colonies grown on the surface of the agar plates supplemented with the active biomaterials after 48 h of incubation. Statistically significant differences were measured when comparing pristine biomaterial and the loaded sample at the same concentration (*** *p* < 0.0001).

**Figure 12:**
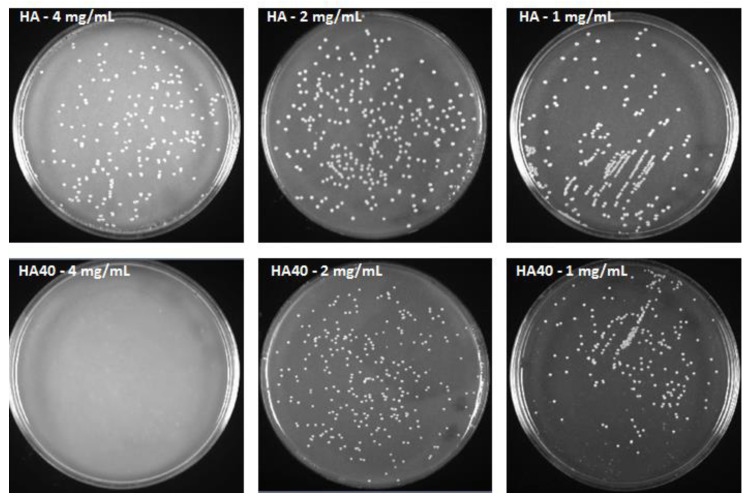
*S. aureus* colonies after 48 h of incubation with HA and HA40 at different concentrations. Colonies grown in presence of HA40 at 2 mg/mL and 1 mg/mL appear smaller than those imaged on the corresponding HA plates.

This assumption was corroborated by the significantly reduced size of the produced colonies. Figure 11 displays the areas of the colonies measured on the surface of the agar plates when supplemented with the active materials compared to the corresponding unloaded samples and to the control agar plate. At 1 mg/mL, CFU were detected on the agar plates starting from the 24 h of incubation, but colonies appeared significantly smaller in size as observed for the higher concentration (Figure 12). The development of smaller colonies at no bactericidal concentrations can be ascribed to the reduced bacterial metabolism; it is widely acknowledged that metal-based materials have multiple antibacterial mechanisms including reactive oxygen species (ROS) generation, inactivation of functional proteins, enzyme system disruption, amino acid metabolism disruption, and prevention of bacterial active transport. Herein, as a bacteriostatic effect was observed even at the lowest concentrations of WO_3_, it is possible to speculate the interaction of the nanoparticles with intra/extracellular molecules involved in the bacterial metabolism [30].

As an in vitro proof-of-concept on the safety of the HA composite biomaterials, in vitro cytotoxicity and cell viability assays were performed on Vero cell line as they are nonmalignant cells with well-defined culturing characteristics in the experimental settings [31,32,33]. Briefly, cells were incubated for 48 h with the different samples; thereafter, their viability was measured and compared to untreated cells. For this purpose, the exposure model system was devised by using PET-transwell inserts and standard 12-well tissue culture plates; cell monolayers were cultured on the tissue plates in regular medium for 24 h, and exposed to biomaterials by adding the samples to the medium in the apical compartment. Data (Figure S5) revealed that only HA120 at the highest concentration slightly reduced cell viability (17 %), while all the other samples did not interfere with mammalian metabolism and proliferation. However, as a sample is considered cytotoxic when its viability is <70% in comparison to untreated cells, none of the HA biomaterials loaded with WO_3_ is cytotoxic, at the used experimental conditions. In addition, LDH assay was performed on the basolateral media, and only a negligible presence of lactate dehydrogenase enzyme (<5%) was measured for HA120 (Figure S6). As the amount of released enzyme from cells is one of the major methods to assess the cell death, the suitability of the proposed materials in the biomedical field was ascertained.

## 4. Conclusions

In this work, we utilized PVP-stabilized WO_3_ nanoparticles to develop composite materials with improved biological properties. The nanoparticles exhibit very small dimensions, and their presence does not affect the structural and morphological properties of the substrates. The attraction of WO_3_ nanoparticles towards different substrates is modulated by their negative zeta potential, so their loaded amount on HAPAA is very small and increases on HA and even more on HAPEI. The strong interaction between the negative nanoparticles and the positively charged HAPEI is confirmed by the smaller cumulative release from HAPEI40 than HA40. In agreement, HA has resulted to be the optimal material to support WO_3_, enabling the diffusion of the active compound at inhibitory concentrations. The analysis with colony-forming units showed an excellent bactericidal activity of HA decorated with WO_3_ nanoparticles against *S. aureus*. In particular, HA40 at 4 mg/mL completely inhibited bacterial growth, in absence of cytotoxicity on the tested mammalian cells. Further biological evaluations as well as in vivo studies will be devised to assess the suitability of this material in biomedical applications. 

## Figures and Tables

**Table 1:** Coherent lengths (*τ_hkl_*) of the perfect crystalline domains in the direction normal to (002) and to (310) planes, and values of zeta potential of the different samples.

Sample	τ 002 (Å)	τ 310 (Å)	Zeta Potential (mV)
HA	510 (10)	255 (10)	−11.3
HA5	514 (12)	262 (8)	−18.9
HA20	518 (13)	258 (10)	−22.5
HA40	513 (10)	266 (15)	−20.3
HA80	508 (15)	258 (10)	−21.1
HA120	490 (18)	262 (12)	−20.7
HAPEI	569 (8)	375 (8)	20.7
HAPEI5	566 (10)	371 (6)	19.2
HAPEI20	577 (8)	367 (8)	12.6
HAPEI40	564 (10)	379 (10)	13.4
HAPAA	460 (10)	204 (12)	−21.0
HAPAA5	454 (15)	218 (12)	−22.8
HAPAA20	470 (15)	218 (13)	−22.4
HAPAA40	455 (12)	199 (15)	−23.8

## Data Availability

The data presented in this study are available on request from the corresponding author.

## Supplementary Materials

The following supporting information can be downloaded at: https://www.mdpi.com/article/10.3390/jfb13030088/s1, Figure S1: Image of composite materials; Figure S2: Size distribution of the PVP-stabilized WO3 nanoparticles; Figure S3: X-ray diffraction patterns of the samples obtained through interaction of HA with increasing volumes of WO3 colloidal suspension; Figure S4: HR-TEM micrographs of HA40, HAPAA40, HAPEI40. In the inset, Fast Fourier Transform of the micrograph; Figure S5: Viability of Vero cells incubated with the composite materials for 48 h.; Figure S6: LDH results obtained in Vero cells incubated with the composite materials for 48 h.

## References

[1] Tande A.J., Palraj B.R., Osmon D.R., Berbari E.F., Baddour L.M., Lohse C.M., Steckelberg J.M., Wilson W.R., Sohail M.R. (2016). Clinical presentation, risk factors, and outcomes of hematogenous prosthetic joint infection in patients with staphylococcus aureus bacteremia. Am. J. Med..

[2] Duan G., Chen L., Jing Z., De Luna P., Wen L., Zhang L., Zhao L., Xu J., Li Z., Yang Z. (2019). Robust antibacterial activity of tungsten oxide (WO_3-x_) nanodots. Chem. Res. Toxicol..

[3] Deshmukh S.P., Patil S.M., Mullani S.B., Delekar S.D. (2019). Silver nanoparticles as an effective disinfectant: A review. Mater. Sci. Eng. C.

[4] Ducel G., Fabry J., Nicolle L. (2002). Prevention of Hospital-Acquired Infections: A Practical Guide.

[5] Tang S., Zheng J. (2018). Antibacterial activity of silver nanoparticles: Structural effects. Adv. Healthc. Mater..

[6] Surmeneva M., Lapanje A., Chudinova E., Ivanova A., Koptyug A., Loza K., Prymak O., Epple M., Ennen-Roth F., Ulbricht M. (2019). Decreased bacterial colonization of additively manufactured Ti6Al4V metallic scaffolds with immobilized silver and calcium phosphate nanoparticles. Appl. Surf. Sci..

[7] Stavitskaya A., Shakhbazova C., Cherednichenko Y., Nigamatzyanova L., Fakhrullina G., Khaertdinov N., Kuralbayeva G., Filimonova A., Vinokurov V., Fakhrullin R. (2020). Antibacterial properties and in vivo studies of tannic acid-stabilized silver–halloysite nanomaterials. Clay Miner..

[8] AshaRani P.V., Low Kah Mun G., Hande M.P., Valiyaveettil S. (2009). Cytotoxicity and genotoxicity of silver nanoparticles in human cells. ACS Nano.

[9] Chen Z., Meng H., Xing G., Chen C., Zhao Y., Jia G., Wang T., Yuan H., Ye C., Zhao F. (2006). Acute toxicological effects of copper nanoparticles in vivo. Toxicol. Lett..

[10] Haick H., Paz Y. (2003). Long-range effects of noble metals on the photocatalytic properties of titanium dioxide. J. Phys. Chem. B.

[11] Muzaffar T., Khosa R.Y., Iftikhar U., Obodo R.M., Sajjad S., Usman M. (2021). Synthesis and characterization of WO_3_/GO nanocomposites for antimicrobial properties. J. Clust. Sci..

[12] Zheng H., Ou J.Z., Strano M.S., Kaner R.B., Mitchell A., Kalantar-zadeh K. (2011). Nanostructured tungsten oxide—Properties, synthesis, and applications. Adv. Funct. Mater..

[13] Wen L., Chen L., Zheng S., Zeng J., Duan G., Wang Y., Wang G., Chai Z., Li Z., Gao M. (2016). Ultrasmall biocompatible WO_3-x_ nanodots for multi-modality imaging and combined therapy of cancers. Adv. Mater..

[14] Salje E.K.H., Rehmann S., Pobell F., Morris D., Knight K.S., Herrmannsdorfer T., Dove M.T. (1997). Crystal structure and paramagnetic behaviour of ε-WO_3−x_. J. Phys. Condens. Mater..

[15] Vogt T., Woodward P.M., Hunter B.A. (1999). The high-temperature phases of WO_3_. J. Solid State Chem..

[16] Popov A.L., Han B., Ermakov A.M., Savintseva I.V., Ermakova O.N., Popova N.R., Shcherbakov A.B., Shekunova T.O., Ivanova O.S., Kozlov D.A. (2020). PVP-stabilized tungsten oxide nanoparticles: pH sensitive anti-cancer platform with high cytotoxicity. Mater. Sci. Eng. C.

[17] Matharu R.K., Ciric L., Ren G., Edirisinghe M. (2020). Comparative study of the antimicrobial effects of tungsten nanoparticles and tungsten nanocomposite fibres on hospital acquired bacterial and viral pathogens. Nanomaterials.

[18] Bigi A., Boanini E. (2018). Calcium phosphates as delivery systems for bisphosphonates. J. Funct. Biomater..

[19] Boanini E., Torricelli P., Cassani M.C., Gentilomi G.A., Ballarin B., Rubini K., Bonvicini F., Bigi A. (2014). Cationic-anionic polyelectrolyte interaction as a tool to graft silver nanoparticles on hydroxyapatite crystals and prevent cytotoxicity. RSC Adv..

[20] Boanini E., Cassani M.C., Rubini K., Boga C., Bigi A. (2018). (9R)-9-Hydroxystearate-functionalized anticancer ceramics promote loading of silver nanoparticles. Nanomaterials.

[21] Boanini E., Torricelli P., Cassani M.C., Rubini K., Fini M., Pagani S., Bigi A. (2020). Platinum nanoparticles supported on functionalized hydroxyapatite: Anti-oxidant properties and bone cells response. Ceram. Int..

[22] (2015). Methods for Dilution Antimicrobial Susceptibility Tests for Bacteria That Grow Aerobically.

[23] Bonvicini F., Manet I., Belluti F., Gobbi S., Rampa A., Gentilomi G.A., Bisi A. (2019). Targeting the bacterial membrane with a new polycyclic privileged structure: A powerful tool to face *Staphylococcus aureus* infections. ACS Infect. Dis..

[24] Kozlov D.A., Shcherbakov A.B., Kozlova T.O., Angelov B., Kopitsa G.P., Garshev A.V., Baranchikov A.E., Ivanova O.S., Ivanov V.K. (2020). Photochromic and photocatalytic properties of ultra-small PVP-stabilized WO_3_ nanoparticles. Molecules.

[25] Bigi A., Boanini E., Gazzano M., Kojdecki M.A., Rubini K. (2004). Microstructural investigation of hydroxyapatite-polyelectrolyte composites. J. Mater. Chem..

[26] Forte L., Sarda S., Combes C., Brouillet F., Gazzano M., Marsan O., Boanini E., Bigi A. (2017). Hydroxyapatite functionalization to trigger adsorption and release of risedronate. Colloids Surf. B Biointerfaces.

[27] Nikaido H. (2003). Molecular basis of bacterial outer membrane permeability revisited. Microbiol. Mol. Biol. Rev..

[28] Cox G., Wright G.D. (2013). Intrinsic antibiotic resistance: Mechanisms, origins, challenges and solutions. Int. J. Med. Microbiol..

[29] Ismail A.S., Tawfik S.M., Mady A.H., Lee Y.-I. (2021). Preparation, properties, and microbial impact of tungsten (VI) oxide and zinc (II) oxide nanoparticles enriched polyethylene sebacate nanocomposites. Polymers.

[30] Slavin Y.N., Asnis J., Häfeli U.O., Bach H. (2017). Metal nanoparticles: Understanding the mechanisms behind antibacterial activity. J. Nanobiotechol..

[31] (2009). Biological Evaluation of Medical Devices—Part 5: Tests for In Vitro Cytotoxicity.

[32] Rodrigues A.A., Batista N.A., Malmonge S.M., Casarin S.A., Agnelli J.A.M., Santos A.R., Belangero W.D. (2021). Osteogenic differentiation of rat bone mesenchymal stem cells cultured on poly (hydroxybutyrate-co-hydroxyvalerate), poly (ε-caprolactone) scaffolds. J. Mater. Sci. Mater. Med..

[33] Mohonta S.K., Maria K.H., Rahman S., Das H., Hoque S.M. (2021). Synthesis of hydroxyapatite nanoparticle and role of its size in hydroxyapatite/chitosan–gelatin biocomposite for bone grafting. Int. Nano Lett..

