# Supplementary material for: Hydroxyapatite Decorated with Tungsten Oxide Nanoparticles: New Composite Materials against Bacterial Growth
Source: J Funct Biomater. 2022 Jun 24;13(3):88. doi: 10.3390/jfb13030088 (PMC9326691; doi:10.3390/jfb13030088)
Supplement: Supplementary file 1 [file jfb-13-00088-s001.zip › jfb-1761072-SI.pdf]

Supplementary Material

Hydroxyapatite Decorated with Tungsten Oxide Nanoparticles: New Composite Materials against Bacterial Growth

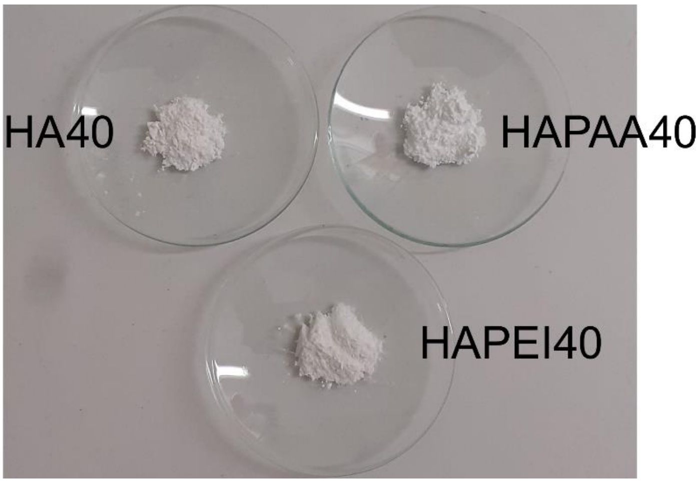

Figure S1. Image of composite materials.

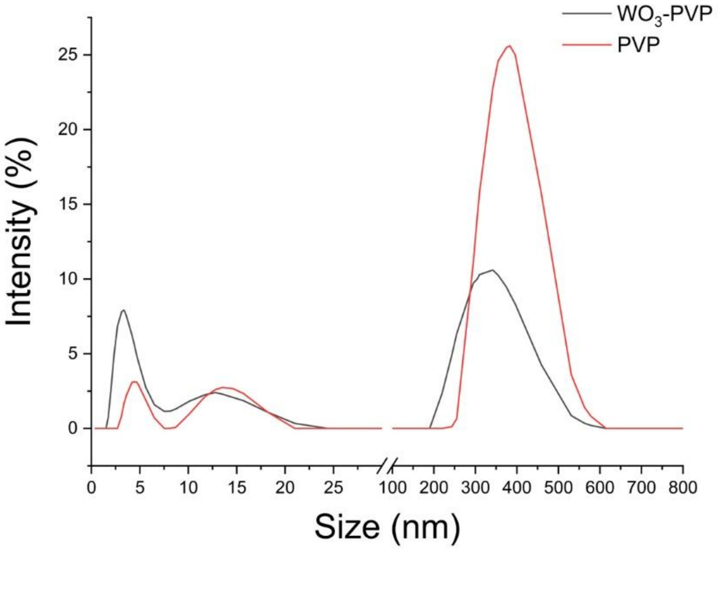

Figure S2. Size distribution of the PVP-stabilized WO_3 nanoparticles.

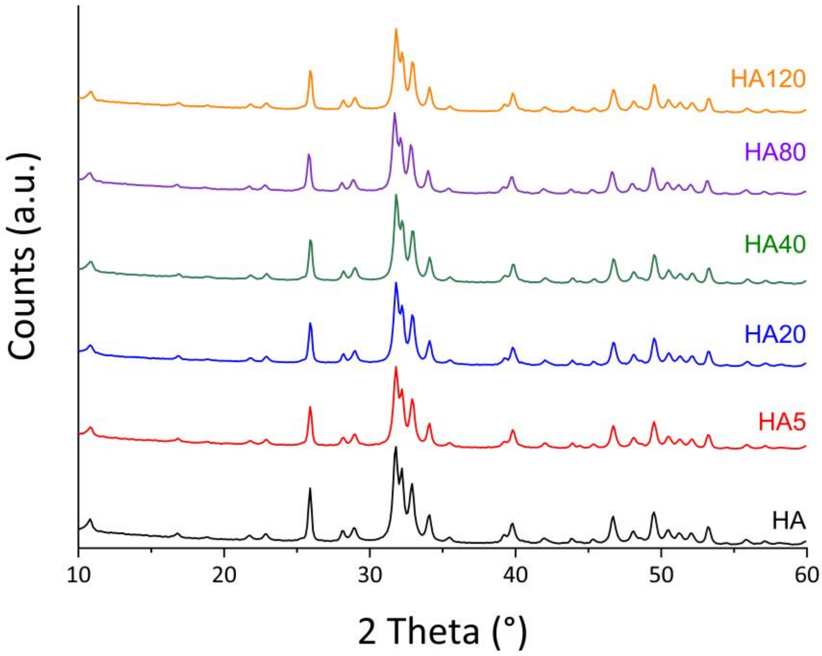

Figure S3. X-ray diffraction patterns of the samples obtained through interaction of HA with increasing volumes of WO_3 colloidal suspension.

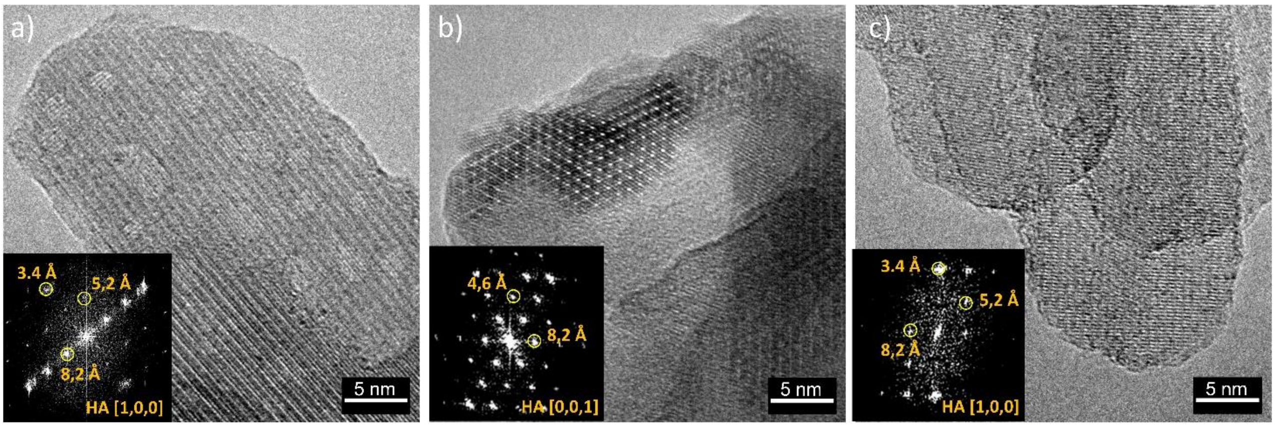

Figure S4. HR-TEM micrographs of (**a**) HA40, (**b**) HAPAA40, (**c**) HAPEI40. In the inset, Fast Fourier Transform of the micrograph displaying the typical pattern resulting from Hydroxyapatite structure. Zone axis orientation and d-spacing corresponding to the main spots overlayed.

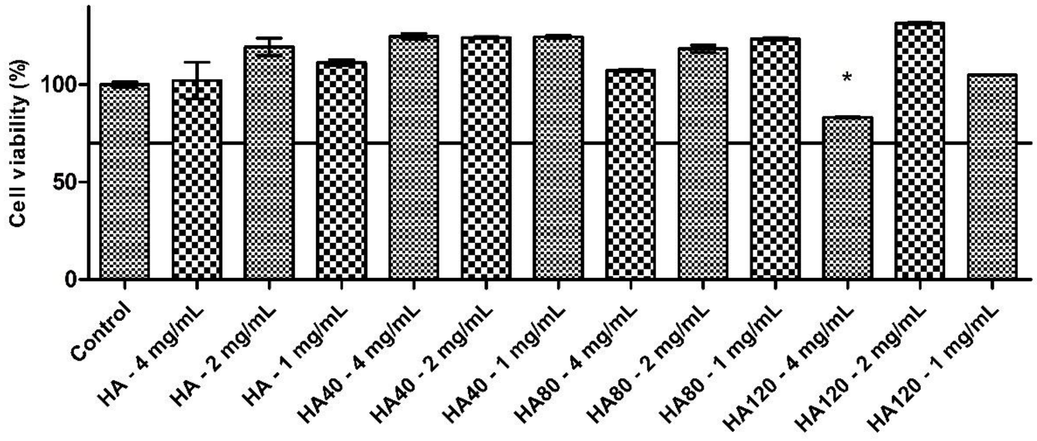

Figure S5. Viability of Vero cells incubated with the composite materials for 48 h. Values are means with standard deviations and are relative to the untreated control (100% of growth). The horizontal line is set at 70% indicating the threshold of the cytotoxicity. A significant decrease in viability was observed for cells incubated with HA120 at 4 mg/mL in comparison with HA, HA40 and HA80 at the same concentration (*p<0.05).)

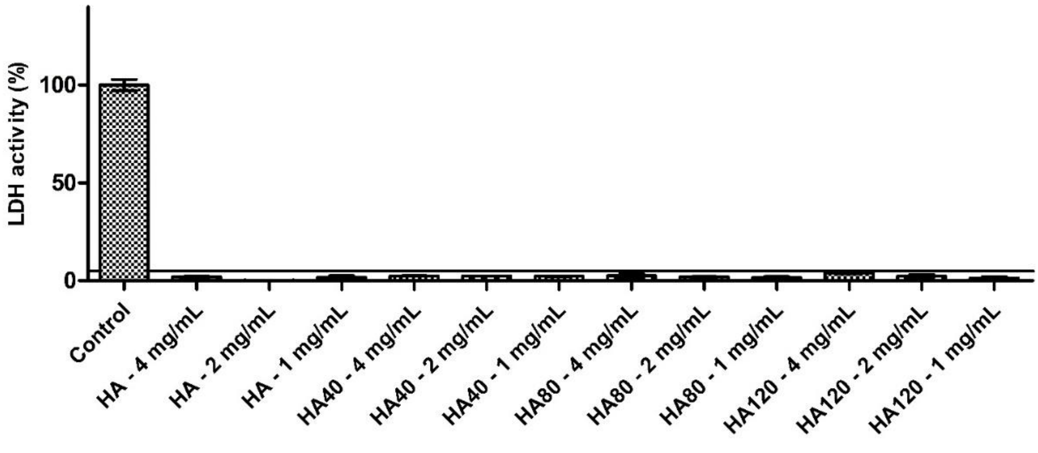

Figure S6. LDH results obtained in Vero cells incubated with the composite materials for 48 h. Values are means with standard deviations and are relative to the positive control (lysed cells). The horizontal line is arbitrarily set at 5%. None of the tested biomaterials induced cell death.
